# HIV and COVID-19 pandemic collision: turning challenges into opportunity

**DOI:** 10.2217/fvl-2020-0382

**Published:** 2021-04-22

**Authors:** Stefano Rusconi, Laura Pezzati, Tiziana Formenti, Andrea Giacomelli

**Affiliations:** 1 ^1^III Infectious Disease Unit, ASST-FBF-Sacco, Milan, Italy; 2 ^2^Department of Biomedical & Clinical Sciences DIBIC L Sacco, University of Milan, Milan, Italy

**Keywords:** HIV, missed opportunities, outcomes, pandemic, retention in care, SARS-CoV-2

Since the first onset of the SARS-CoV-2 pandemic in China, it clearly appeared that the healthcare systems around the world would be severely challenged, in particular regarding their capability to handle patients with chronic conditions [1]. HIV infection is among these and the continuum of care such as the availability of diagnostic and preventive resources are particularly important to combat the HIV pandemic. In fact, multifaceted strategies are required to curb the HIV pandemic and to approach the ambitious goal of 90-90-90 [2]. In particular, these goals include that by 2020, 90% of people living with HIV will be aware of their status, 90% of people diagnosed will receive antiretrovirals and 90% of people on therapy will have their HIV-RNA under control. The 90-90-90 strategy relies on HIV diagnosis and prevention which requires the availability of outpatients’ facilities to perform HIV screening and to give counseling and distribute pre-exposure prophylaxis. Moreover, for people living with HIV and AIDS (PLWHA) the continuum of care is one of the key points to maintain the treatment and consolidate the so called ‘third 90’ [2].

As the two pandemics collided, several concerns on HIV diagnosis and management have been raised (Supplementary Figure 1).

First, the measures enforced by most of the countries to reduce the spread of SARS-CoV-2 comprehended national or local lockdowns and restriction on travel between countries [3]. This type of confinement, if on the one hand has been demonstrated to be the most effective strategy, able to reduce the burden and diffusion of SARS-CoV-2, on the other hand, it creates more barriers for patients with chronic diseases such as those PLWHA [4]. Moreover, most of the outpatients’ facilities were temporary closed and consequently both HIV new diagnosis [5], postexposure prophylaxis [6,7] and pre-exposure prophylaxis dropped due to healthcare resources re-allocated to fight the COVID-19 pandemic [8].

Second, contradicting evidence from the possible therapeutic effect of some antiretrovirals (i.e., lopinavir/ritonavir) came to public attention in the early days of the pandemic. These evidences were subsequently disproved by several studies, which showed no benefit on patient survival [9,10]. Nevertheless, contradictory messages coming from the media suggested the wrong message, in other words, PLWHA on treatment with such medications could have a decreased risk of disease severity [11].

Third, the emergence of a viral disease which affected the most fragile groups, such as elderly people and those with underlying medical conditions, added fear to PLWHA about a possibly more severe course of the diseases related to the immunosuppression. Nevertheless, it seems that PLWHA do not show a more severe phenotype of the disease since the first preliminary reports [12]. These observations were subsequently confirmed by large observational studies which identified age and comorbidities as the most important drivers toward a worse outcome in PLWHA [13,14]. As far as HIV specific immunological deficiency, those patients without antiretroviral treatment, such as those with lower CD4 cell counts, appeared to be at increased risk of severe disease and worse outcomes [15]. For all these reasons and to ensure an ethical equipoise to our patients, as many as possible PLWHA should be included in clinical trials of anti-COVID-19 drugs.

The well-known problem of double stigma that was well known in the intersecting TB and HIV pandemics [16] is now renewed in the HIV and COVID-19 collision [4]. In particular, cultural and gender minorities are those at increased risk to be left behind when the stigma for one disease (i.e., HIV) is increased by the fear and stigma from another one (i.e., COVID-19) [17]. The COVID-19 pandemic is highly impacting on nonwhite people, illegal immigrants and in general on people who are economically disadvantaged, who are already deeply hit by the HIV epidemic. This double stigma can be a serious hurdle for the HIV care continuum, making it impossible to maintain an optimal antiretroviral adherence and thus jeopardizing the 90-90-90 strategy.

Even if several barriers to diagnosis and care have arisen during the current pandemic, we should not miss the opportunity to turn the challenges highlighted by the pandemic into an opportunity to look with renewed interest at the innovation of care of PLWHA [18]. The first 90 regards the issue of PLWHA left without a diagnosis. This is a serious problem highlighted by the high rate of missed opportunity of diagnosis and the high proportion of the so called ‘late presenter’ among the new HIV diagnosis [19]. A huge opportunity during the COVID-19 pandemic should be to increase the knowledge of HIV self-testing, particularly where the access to outpatients’ clinics are difficult [20]. Moreover, the access to healthcare due to SARS-CoV-2 infection could also be an opportunity to screen for other communicable diseases such as viral hepatitis and TB. In regards to the ‘second and third 90’, it is time to take the opportunity to implement two main aspects: telehealth and HIV medicines access [18]. Telehealth would be able to reduce barriers to care by removing the requirements of transportation and stigma. In particular, research in this field would help to determine the right balance between ‘*de visu’* evaluations and remote ones. The problem of drug access is a serious concern in some remote and rural areas of the world and the COVID-19 pandemic has also created further barriers to pick up medicines in high-resource settings. Several strategies would be able to overcome the current barrier to drug dispensing such as: extra supply of medications; possibility to pick up medicines in nonhospital pharmacies; and home delivery. Another strategy is represented by the administration of long-acting compounds like cabotegravir (CAB) and rilpivirine (RPV), since they did not exhibit a huge impact by COVID-19. In a review of data from six ongoing trials with the investigational agents CAB + RPV longacting, 93% of participants maintained their injection visits, with no virologic failures or development of resistance. The few participants with missed injection visits were maintained on continuous antiretroviral therapy by being temporarily switched to oral therapy, most often with oral CAB + RPV or an alternative oral antiretroviral combination, which underlines the feasibility of bridging between intramuscular and oral therapy [21].

Last but not least, COVID-19 vaccines – at least four of them – can be viewed as a parallel effort, which could involve PLWHA.

In conclusion, the COVID-19 pandemic is severely challenging the care of people living with chronic diseases such as PLWHA. Renewed challenges are growing up such as the continuum of care for PLWHA and the worrisome problem of double stigma. Nevertheless, the COVID-19 pandemic is accelerating several aspects of science and new opportunities of innovative care strategies for PLWHA. As physicians involved in the care of PLWHA, we have now a duty to make an extra effort to turn the current challenges into new opportunities.
