# Supplementary material for: HIV and COVID-19 pandemic collision: turning challenges into opportunity
Source: Future Virol. 2021 Apr 22:10.2217/fvl-2020-0382. doi: 10.2217/fvl-2020-0382 (PMC8061429; doi:10.2217/fvl-2020-0382)
Supplement: Supplementary file 1 [file supplementary_figure_1.pdf]

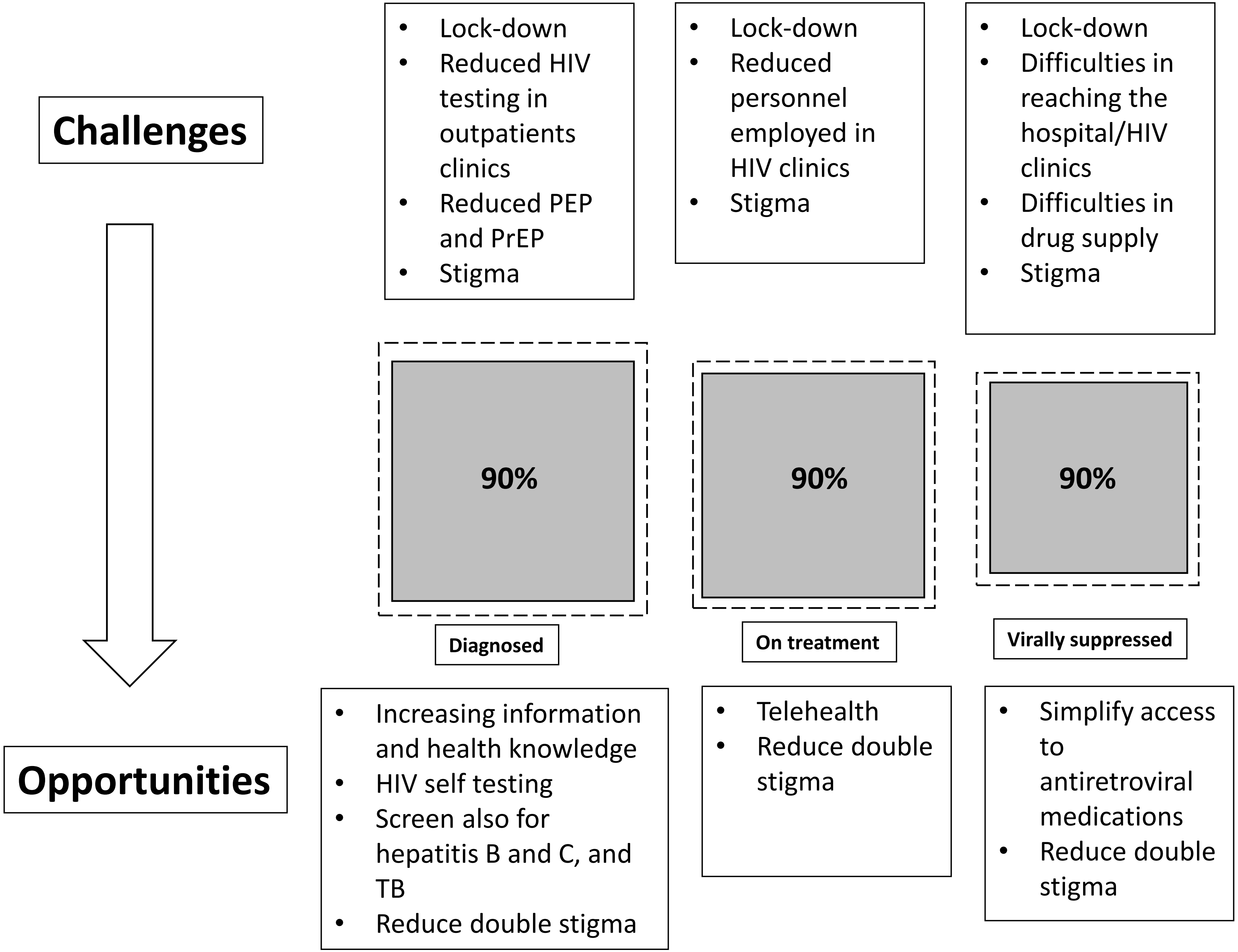
Challenges
• Lock-down
• Reduced HIV testing in outpatients clinics
• Reduced PEP and PrEP
• Stigma
• Lock-down
• Reduced personnel employed in HIV clinics
• Stigma
• Lock-down
• Difficulties in reaching the hospital/HIV clinics
• Difficulties in drug supply
• Stigma
90%
90%
90%
Diagnosed
On treatment
Virally suppressed
Opportunities
• Increasing information and health knowledge
• HIV self testing
• Screen also for hepatitis B and C, and TB
• Reduce double stigma
• Telehealth
• Reduce double stigma
• Simplify access to antiretroviral medications
• Reduce double stigma
